# Intestinal L-cell mechanoreception regulates hepatic lipid metabolism through GLP-1

**DOI:** 10.1126/sciadv.adv3201

**Published:** 2025-05-30

**Authors:** Luyang Gao, Ke Yang, Yawen Zhao, Jinshan Zhang, Shaohua Jiang, Rujiao Zhang, Wenxin He, Yuhang Zhao, Qianqian Ye, Geyang Xu

**Affiliations:** ^1^ Department of Physiology, School of Medicine, Jinan University, 601 Huangpu Avenue West, Tianhe District, Guangzhou, Guangdong 510632, China.; ^2^ Key Laboratory of Viral Pathogenesis & Infection Prevention and Control (Jinan University), Ministry of Education, Guangzhou, Guangdong 510632, China.

## Abstract

Glucagon-like peptide–1 (GLP-1), secreted by intestinal L cells, is essential for lowering postprandial glucose levels and regulating hepatic lipid metabolism.We investigate the effects of manipulating Piezo1 in L cells on hepatic lipid metabolism. We found that normal and high-fat diet–fed L cell–specific *Piezo1* knockout (*IntL-Piezo1^−/−^*) mice exhibited reduced circulating GLP-1 levels, increased hepatic lipid accumulation, decreased β-catenin expression, and elevated lipogenesis-related genes and proteins, including SREBP1c, PPARγ, FASN, and ACC. Treatment with exendin-4 improved fatty liver in *IntL-Piezo1^−/−^* mice by stimulating β-catenin and inhibiting de novo lipogenesis. Intestinal bead implantation stimulated GLP-1 release and inhibited lipid synthesis in livers of diet-induced obese mice but not in *IntL-Piezo1^−/−^* mice. In primary hepatocytes derived from *IntL-Piezo1^−/−^* mice, lipid accumulation and enhanced fatty acid synthesis were associated with reduced β-catenin expression and impaired nuclear translocation. Exendin-4 treatment alleviated lipid accumulation, which was blocked by the β-catenin inhibitor nitazoxanide. L-cell mechanoreception is vital for regulating hepatic lipid metabolism through GLP-1.

## Teaser

L-cell mechanoreception plays a key role in regulating liver lipid metabolism via GLP-1.

## INTRODUCTION

Metabolic dysfunction–associated steatotic liver disease (MASLD) is a prevalent liver disease characterized by excessive fat accumulation in the liver. Its prevalence rises alongside obesity and metabolic disorders. Chronic fat accumulation can progress from simple fatty liver (nonalcoholic fatty liver) to more severe forms of steatohepatitis (nonalcoholic steatohepatitis), potentially advancing to liver fibrosis, cirrhosis, and even liver cancer (*1*–*4*). The current management of MASLD primarily focuses on lifestyle modifications, such as adopting a low-calorie diet, engaging in regular physical activity, and achieving weight loss (*5*). In addition, certain hypoglycemic agents show promise in the treatment of MASLD, including peroxisome proliferator–activated receptor (PPAR) agonists, sodium-glucose cotransporter 2 inhibitors, and glucagon-like peptide–1 receptor (GLP-1R) agonists (*6*). Glucagon-like peptide–1 (GLP-1) is a hormone primarily secreted by intestinal L cells and is considered a promising candidate for the treatment of MASLD (*7*). Previous studies have demonstrated that GLP-1 has a variety of physiological properties, including enhanced insulin secretion (*8*), reduced appetite (*9*), promotion of adipose differentiation (*10*), and inhibition of hepatic lipogenesis (*11*). Pharmacological interventions aimed at GLP-1 and its receptor have demonstrated the ability to slow the progression of hepatic steatosis related to obesity (*12*, *13*). Therefore, modulating GLP-1 production may provide a promising strategy to improve hepatic lipid metabolism.

Piezo1 is a mechanosensitive ion channel that responds to mechanical forces such as tension (*14*) and shear (*15*, *16*). This channel is essential for various physiological processes, including touch sensation (*17*), blood pressure regulation (*18*), and the development and progression of chronic inflammatory diseases (*19*). Piezo1 is expressed in intestinal epithelial cells and regulates intestinal motility (*20*), barrier function (*21*), and mucus secretion (*22*). Our previous study demonstrated that the mechanosensing properties of Piezo1 in intestinal L cells are essential for regulating GLP-1 production and glucose metabolism (*23*). Building on this discovery, we explored the role of intestinal Piezo1 in regulating hepatic lipid metabolism using L cell–specific *Piezo1* gene knockout mice (*IntL-Piezo1^−/−^*). Our findings revealed that both low-fat and high-fat diet (HFD)–fed *IntL-Piezo1^−/−^* mice exhibited decreased circulating GLP-1 levels and increased hepatic lipogenesis. Furthermore, we showed that Piezo1 channels in intestinal L cells inhibit hepatic lipid synthesis via GLP-1 signaling. These results offer previously unidentified insights into potential treatments for MASLD.

## RESULTS

### *IntL-Piezo1^−/−^* mice exhibit reduced circulating GLP-1 levels and hepatic steatosis

**Fig. 1.**
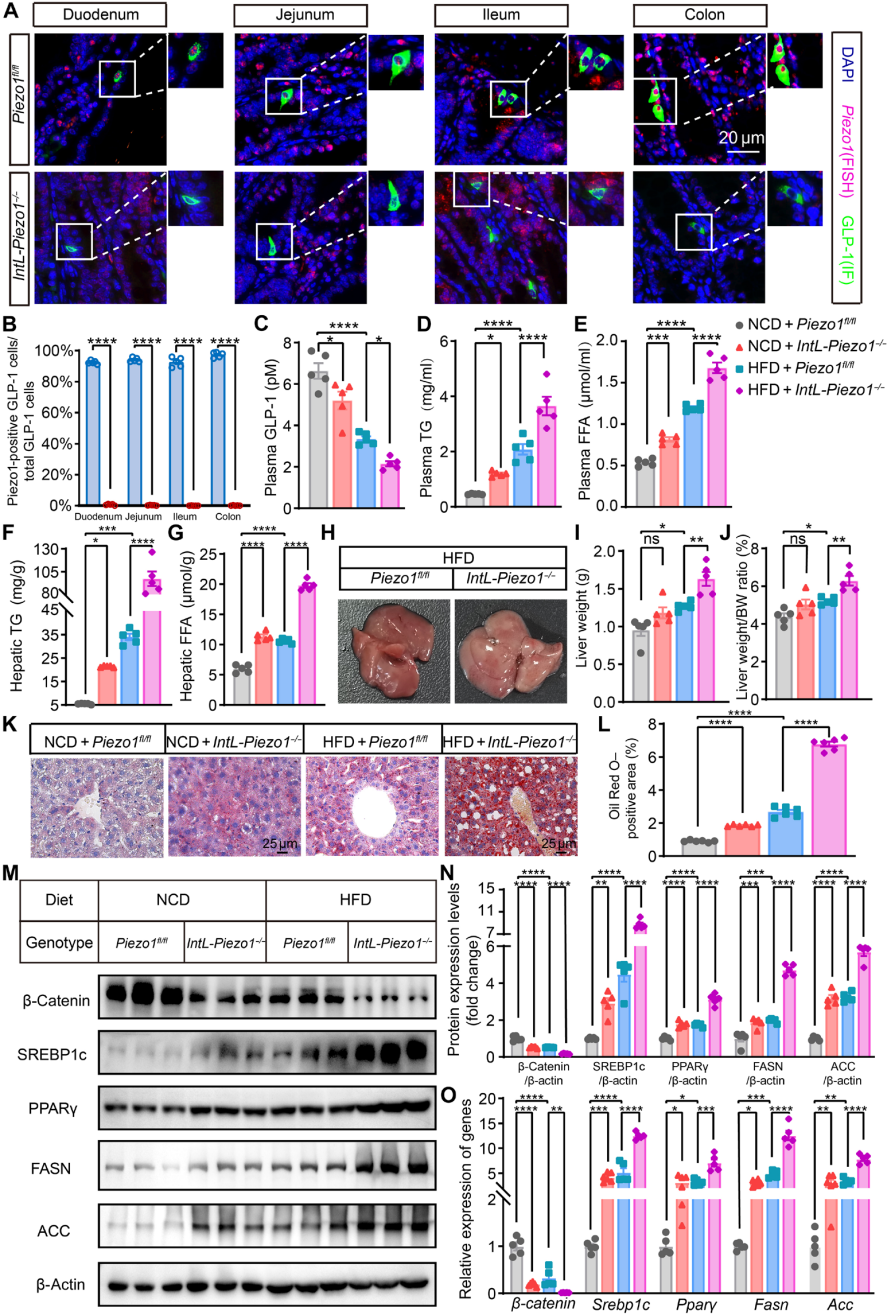
*IntL-Piezo1^−/−^* mice exhibit decreased circulating GLP-1 levels and hepatic steatosis. (**A** and **B**) Representative images showing *Piezo1* RNA fluorescence in situ hybridization (FISH; red) and GLP-1 (green) immunofluorescence (IF) staining in the duodenum, jejunum, ileum, and colon of 14-week-old male *Piezo1^fl/fl^* and *IntL-Piezo1^−/−^* mice (*n* = 5 per group) with quantification. (**C**) Plasma GLP-1 (*n* = 5 per group), (**D**) plasma TG, and (**E**) plasma FFA levels in 14- to 16-week-old male *Piezo1^fl/fl^* and *IntL-Piezo1^−/−^* fed either an NCD or HFD (*n* = 5 per group). (**F** and **G**) Quantification of hepatic levels of TG and FFA in *Piezo1^fl/fl^* and *IntL-Piezo1^−/−^* mice (*n* = 5 per group). (**H**) Evaluation of liver gross morphology, (**I**) liver weight, and (**J**) liver weight–to–body weight (BW) ratio in *Piezo1^fl/fl^* and *IntL-Piezo1^−/−^* (*n* = 5 per group). (**K**) Oil Red O staining of the liver and (**L**) Oil Red O quantitative analysis (*n* = 6 per group). (**M**) Western blot analysis to detect the expression of β-catenin, SREBP1c, PPARγ, FASN, and ACC in the livers of *Piezo1^fl/fl^* and *IntL-Piezo1^−/−^* mice. (**N**) Quantification of Western blot results. (**O**) Hepatic mRNA levels of β*-catenin*, *Srebp1c*, *Ppar*γ, *Fasn*, and *Acc* (*n* = 5 per group). Data in (A) to (O) are presented as the means ± SEM from five or six biological replicates. One-way ANOVA followed by post hoc analysis was used to determine statistical significance in (A) to (O). *P < 0.05, **P < 0.01, ***P < 0.001, and ****P < 0.0001.

To investigate the impact of intestinal L cell–specific *Piezo1* deletion on hepatic lipid accumulation, *Piezo1^fl/fl^* mice were crossed with *IntL-Cre* mice to generate intestinal L cell–specific *Piezo1* knockout strains (*IntL-Piezo1^−/−^*). In situ hybridization analysis of *Piezo1* and GLP-1 revealed that *Piezo1* expression was absent in GLP-1–positive cells of the duodenum, jejunum, ileum, and colon in *IntL-Piezo1^−/−^* mice. This finding confirms the successful knockout of *Piezo1* in the L cells of these mice (Fig. 1, A and B).

Under both normal and high-fat feeding conditions, circulating levels of GLP-1 were significantly lower in *IntL-Piezo1^−/−^* mice compared to control mice, while blood and liver lipid levels were elevated (Fig. 1, C to G). Furthermore, the livers of *IntL-Piezo1^−/−^* mice, particularly when exposed to an HFD, displayed enlargement, a yellow-white appearance, and an increase in liver weight, along with a higher liver weight–to–body weight ratio. In contrast, there were no notable differences in liver weight or liver weight–to–body weight ratio were noted in normal chow diet (NCD)–fed *IntL-Piezo1^−/−^* mice compared to NCD-fed *Piezo1^fl/fl^* mice (Fig. 1, H to J). Moreover, *IntL-Piezo1^−/−^* mice displayed more severe hepatic lipid accumulation than *Piezo1^fl/fl^* mice under both normal and high-fat feeding conditions (Fig. 1, K and L).

We further investigated the expression levels of key genes and proteins involved in fatty acid synthesis. In accordance with the observed changes in hepatic lipid levels, we found that the expression of transcription factors and enzymes related to fatty acid synthesis, such as sterol regulatory element–binding protein 1c (SREBP1c), PPARγ, fatty acid synthase (FASN), and acetyl-CoA carboxylase (ACC), was up-regulated in *IntL-Piezo1^−/−^* mice, with these changes being more pronounced under HFD conditions (Fig. 1, M to O). Previous research has indicated that β-catenin plays a role in regulating hepatic lipid synthesis (*24*). Notably, we observed that the expression levels of β-catenin in the liver were reduced in *IntL-Piezo1^−/−^* mice, regardless of whether they were subjected to an NCD or HFD. This reduction in β-catenin may contribute to the enhanced hepatic lipid synthesis observed in these mice (Fig. 1, M to O).

### Exogenous infusion of exendin-4 improves fatty liver in *IntL-Piezo1^−/−^* mice

**Fig. 2.**
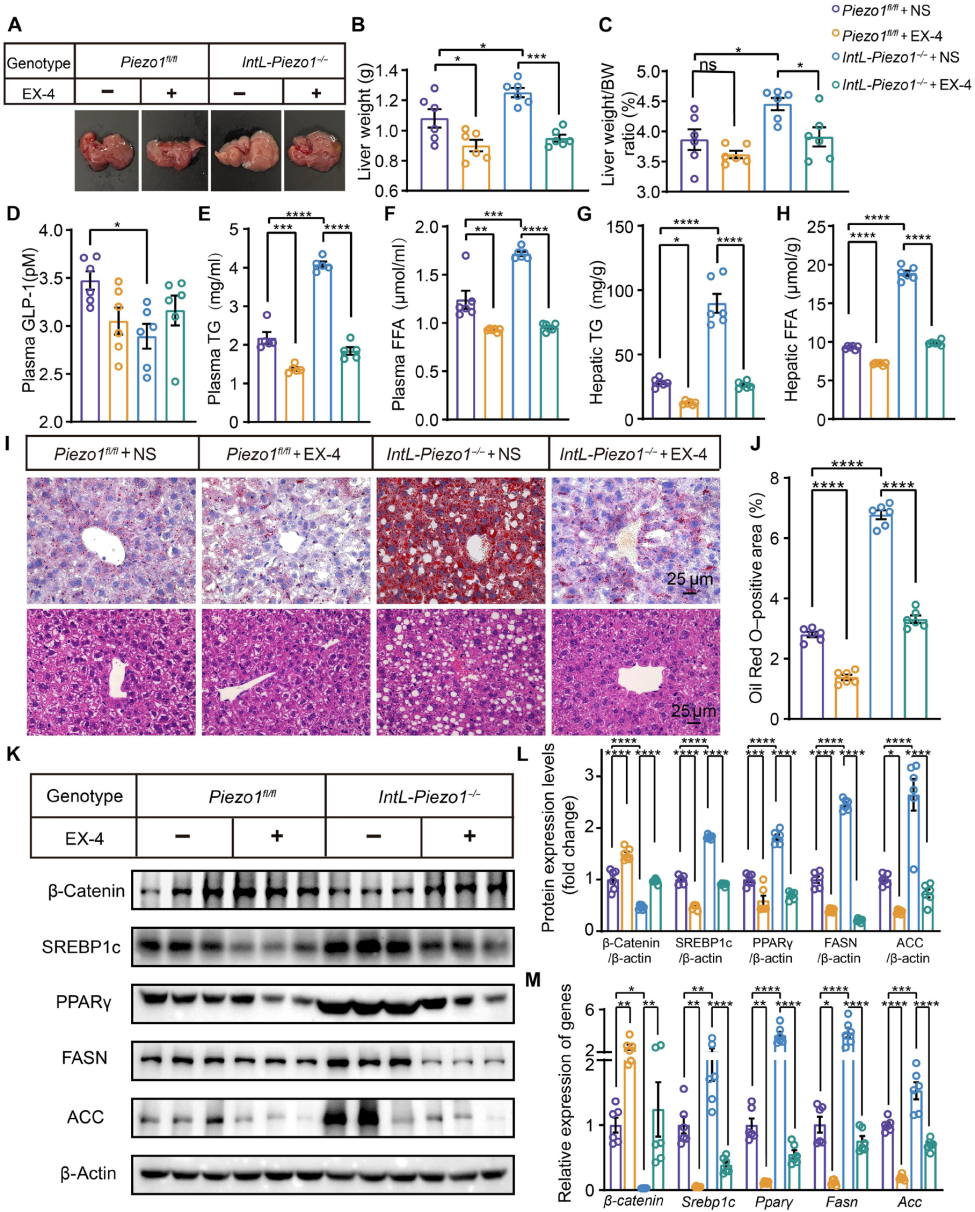
EX-4 relieves hepatic steatosis in *IntL-Piezo1^−/−^* mice under an HFD. (**A**) Representative liver images of *Piezo1^fl/fl^* and *IntL-Piezo1^−/−^* mice after treatment with saline or EX-4. (**B**) Liver weight and (**C**) liver weight–to–body weight ratio of *Piezo1^fl/fl^* and *IntL-Piezo1^−/−^* mice after receiving saline or EX-4 (*n* = 6 per group). (**D**) Plasma GLP-1 levels, (**E**) plasma TG content, (**F**) plasma FFA levels, (**G**) hepatic TG levels, and (**H**) hepatic FFA levels in *Piezo1^fl/fl^* and *IntL-Piezo1^−/−^* mice treated with saline or EX-4 under an HFD (*n* = 6 per group). (**I**) Representative images of hematoxylin and eosin (HE) staining and Oil Red O staining in the livers of *Piezo1^fl/fl^* and *IntL-Piezo1^−/−^* mice treated with saline or EX-4 (*n* = 6 per group). (**J**) Quantification of Oil Red O staining. (**K**) Western blot analysis of β-catenin, SREBP1c, PPARγ, FASN, and ACC in the livers of *Piezo1^fl/fl^* and *IntL-Piezo1^−/−^* mice receiving saline or EX-4 under an HFD (*n* = 6 per group). (**L**) Quantification of Western blot results (*n* = 6 per group). (**M**) Expression levels of lipogenesis-related genes, including β*-catenin*, *Srebp1c*, *Ppar*γ, *Fasn*, and *Acc* (*n* = 6 per group). Data in (A) to (M) are presented as the means ± SEM and represent six biological replicates. Statistical significance was determined using one-way ANOVA followed by post hoc analysis for (A) to (M). *P < 0.05, **P < 0.01, ***P < 0.001, and ****P < 0.0001.

To confirm that hepatic steatosis in *IntL-Piezo1^−/−^* mice is caused by the shortage of GLP-1, we administered exendin-4 (EX-4) intraperitoneally for 7 consecutive days in *Piezo1^fl/fl^* and *IntL-Piezo1^−/−^* mice after 10 weeks of HFD feeding. The results showed that exogenous injection of EX-4 led to a decrease in liver weight and liver weight–to–body weight ratio and circulating lipids, as well as an improvement in hepatic steatosis. However, plasma GLP-1 levels remained unchanged following EX-4 infusion in both *Piezo1^fl/fl^* and *IntL-Piezo1^−/−^* mice (Fig. 2, A to J). In addition, EX-4 inhibited lipid synthesis in the livers of *IntL-Piezo1^−/−^* mice, as evidenced by elevated expression of β-catenin and reduced expression levels of SREBP1c, PPARγ, FASN, and ACC (Fig. 2, K to M).

### Intestinal bead implantation inhibits hepatic lipid accumulation by enhancing GLP-1

**Fig. 3.**
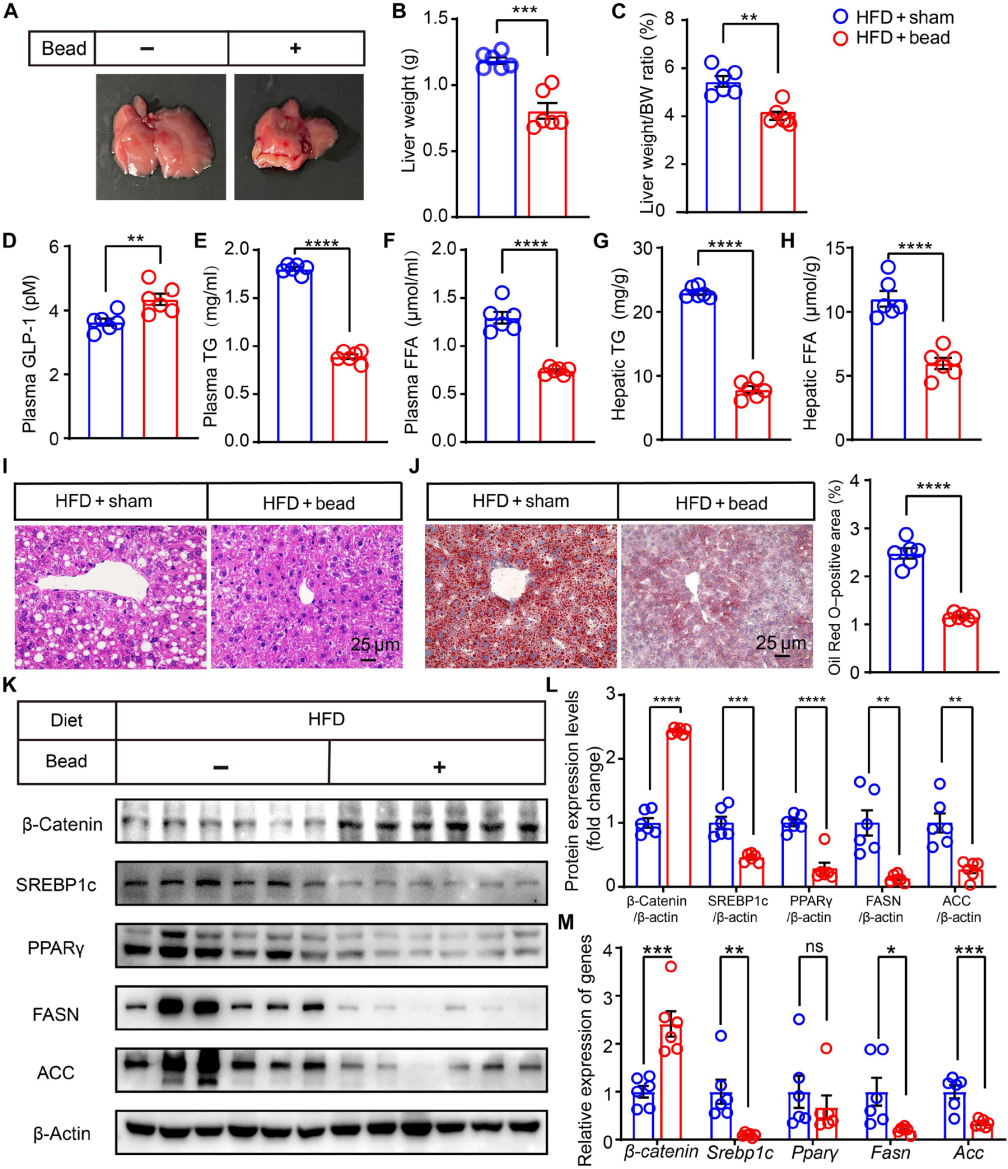
Intestinal bead implantation ameliorates hepatic steatosis in HFD-fed C57BL/6J mice. C57BL/6J mice with diet-induced fatty liver were randomly assigned to either the sham group or intestinal bead implantation group. (**A**) Representative images of the livers from mice in the sham-operated and bead-implanted groups. (**B**) Liver weight and (**C**) liver weight–to–body weight ratio of mice in the sham-operated and bead-implanted groups. (**D**) Levels of plasma GLP-1, (**E**) plasma TG, (**F**) plasma FFA, (**G**) hepatic TG, and (**H**) hepatic FFA in mice from the sham-operated and bead-implanted groups. (**I** and **J**) Representative images of HE staining and Oil Red O staining of the liver tissue. (**K**) Western blot analysis of β-catenin, SREBP1c, PPARγ, FASN, and ACC in the livers of mice in the sham-operated and bead-implanted groups under an HFD. (**L**) Quantification of Western blot results. (**M**) Expression levels of lipogenesis-related genes including β*-catenin*, *Srebp1c*, *Ppar*γ, *Fasn*, and *Acc*. Data in (A) to (M) are presented as the means ± SEM and representative of six biological replicates. Student’s *t* test was used to determine statistical significance in (A) to (M). *n* = 6; *P < 0.05, **P < 0.01, ***P < 0.001, and ****P < 0.0001.

We investigated whether activating Piezo1 could alleviate diet-induced hepatic steatosis in obese mice. After the C57BL/6J mice were fed an HFD for 10 weeks, silicone beads were implanted into their ileum. Our results indicated a significant reduction in liver weight and the liver weight–to–body weight ratio in obese mice following intestinal bead implantation (Fig. 3, A to C). In addition, there was an increase in GLP-1 release (Fig. 3D) alongside a notable decrease in circulating lipids and attenuation of hepatic steatosis in C57BL/6J mice under HFD conditions (Fig. 3, E to J). Intestinal bead implantation significantly up-regulated hepatic β-catenin expression in diet-induced obese mice while inhibiting hepatic lipid synthesis (Fig. 3, K to M). These findings suggest that intestinal mechanical stimulation from intestinal bead implantation enhances GLP-1 production and improves hepatic steatosis.

To further investigate whether the implantation of intestinal silicone beads effectively alleviates fatty liver in L cell–specific *Piezo1* gene knockout mice, we implanted silicone beads into the ileum of *IntL-Piezo1^−/−^* mice after they had been subjected to an HFD for 10 weeks. The results indicated that bead implantation in *IntL-Piezo1^−/−^* mice did not yield the same beneficial effects on fatty liver as observed in C57BL/6J mice with bead implantation. This indicates that intestinal Piezo1 may play a crucial role in this process (fig. S1).

### Primary hepatocytes of *IntL-Piezo1^−/−^* mice show lipid metabolism disorders

**Fig. 4.**
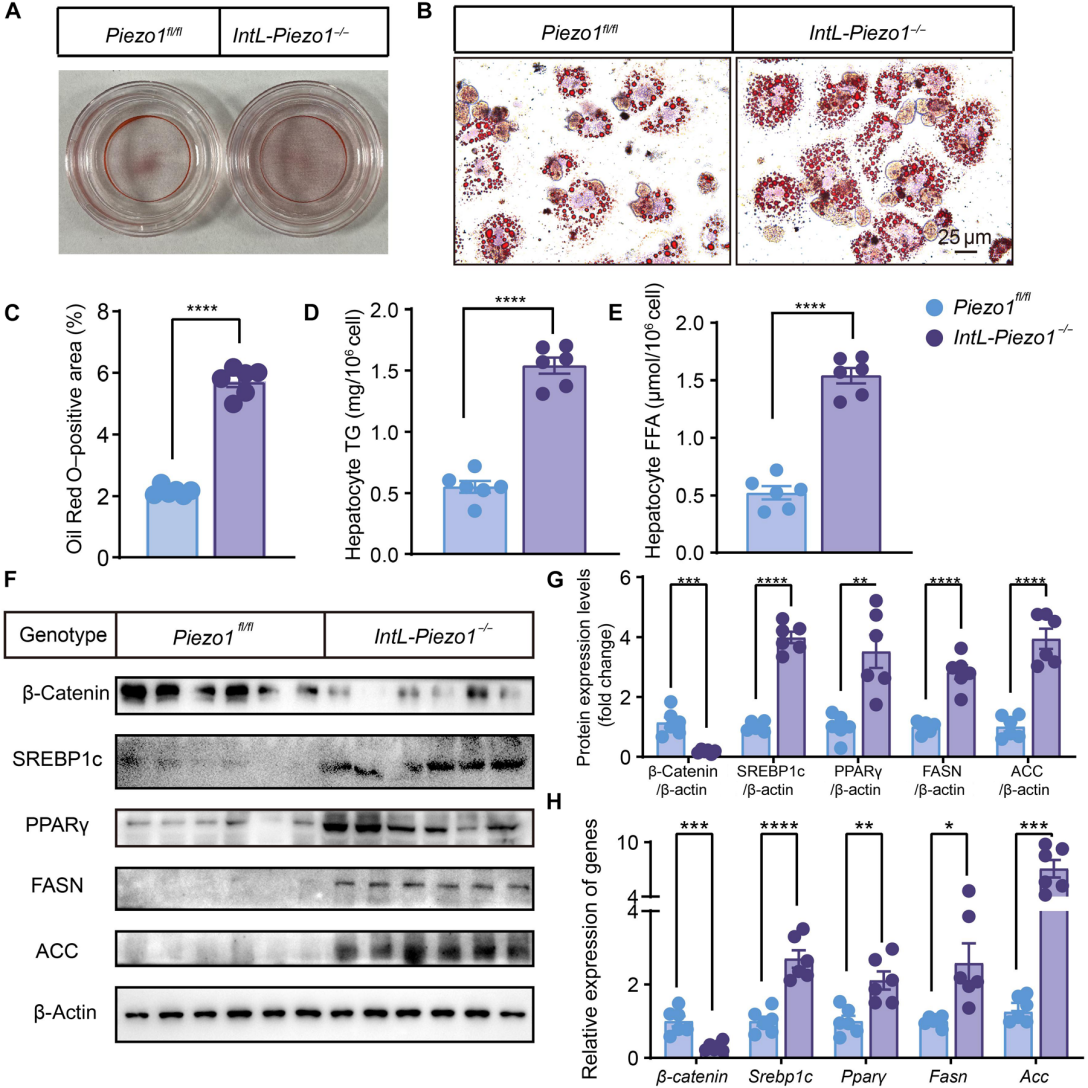
Primary hepatocytes from *IntL-Piezo1^−/−^* mice display abnormalities in lipid metabolism. (**A** and **B**) Oil Red O staining images of primary hepatocytes from *Piezo1^fl/fl^* and *IntL-Piezo1^−/−^* mice under an HFD. (**C**) Quantification of Oil Red O staining. (**D** and **E**) Hepatocyte TG and FFA levels. (**F**) Western blot analysis of whole-cell extracts using antibodies against β-catenin, SREBP1c, PPARγ, FASN, and ACC. (**G**) Quantification of Western blot. (**H**) mRNA levels of β*-catenin*, *Srebp1c*, *Ppar*γ, *Fasn*, and *Acc* in primary hepatocytes from *Piezo1^fl/fl^* and *IntL-Piezo1^−/−^* under high-fat feeding conditions. The data are expressed as the means ± SEM and represent six biological replicates. Statistical significance in (A) to (H) was assessed using Student’s *t* test. *n* = 6; *P < 0.05, **P < 0.01, ***P < 0.001, and ****P < 0.0001.

Primary hepatocytes were isolated from *Piezo1^fl/fl^* and *IntL-Piezo1^−/−^* mice after 10 weeks of high-fat feeding. Compared to hepatocytes from *Piezo1^fl/fl^* mice, those from *IntL-Piezo1^−/−^* mice exhibited significant lipid accumulation (Fig. 4, A to E). This lipid accumulation was associated with the up-regulation of lipogenesis-related transcription factors and enzymes, including SREBP1c, PPARγ, FASN, and ACC (Fig. 4, F to H). Consistent with observations in primary hepatocytes from HFD-fed *IntL-Piezo1^−/−^* mice, primary hepatocytes from NCD-fed *IntL-Piezo1^−/−^* mice also demonstrated lipid accumulation and dysregulated fatty acid metabolism (fig. S2).

### EX-4 reduces lipid accumulation in *IntL-Piezo1^−/−^* hepatocytes by enhancing β-catenin

**Fig. 5.**
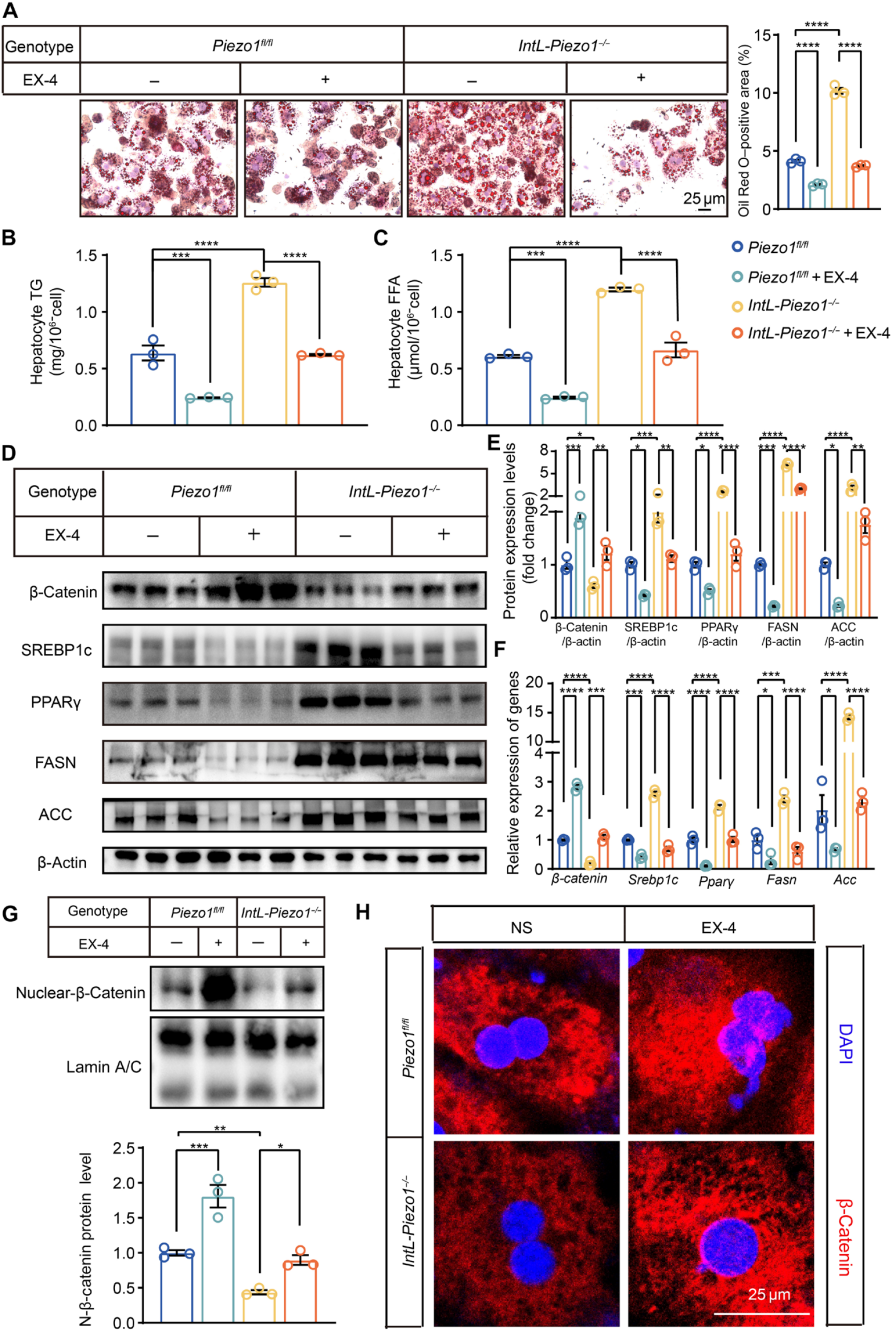
EX-4 ameliorates steatosis in primary hepatocytes of *IntL-Piezo1^−/−^* mice. Oil Red O–stained pictures of primary hepatocytes from HFD-fed *Piezo1^fl/fl^* and *IntL-Piezo1^−/−^* mice after EX-4 (100 nM) treatment for 24 hours. (**A**) Oil Red O staining images of primary hepatocytes. (**B** and **C**) Hepatocyte TG and FFA levels. (**D**) Western blot of whole-cell extracts with the indicated antibodies such as β-catenin, SREBP1c, PPARγ, FASN, and ACC. (**E**) Western blot quantification. (**F**) β*-catenin*, *Srebp1c*, *Ppar*γ, *Fasn*, and *Acc* mRNA levels in primary hepatocytes from HFD-fed *Piezo1^fl/fl^* and *IntL-Piezo1^−/−^* mice after EX-4 treatment for 24 hours. (**G**) Western blot analysis of nuclear extracts was conducted using antibodies such as β-catenin and lamin A/C. The quantification of β-catenin protein was performed accordingly. (**H**) Immunofluorescence staining for β-catenin (red) in primary hepatocytes from HFD-fed *Piezo1^fl/fl^* and *IntL-Piezo1^−/−^* mice after EX-4 treatment for 24 hours. Data are presented as the means ± SEM and represent three biological replicates. Significance was determined by one-way ANOVA followed by post hoc analysis in (A) to (H). *P < 0.05, **P < 0.01, ***P < 0.001, and ****P < 0.0001.

EX-4 reduced hepatic lipid deposition, increased the expression and nuclear translocation of β-catenin, and decreased the protein and mRNA expression levels of lipogenesis-related transcription factors and enzymes (such as SREBP1c, PPARγ, FASN, and ACC) in primary hepatocytes derived from HFD-fed *Piezo1^fl/fl^* and *IntL-Piezo1^−/−^* mice (Fig. 5, A to H). Consistent with the observations in primary hepatocytes from HFD-fed mice, primary hepatocytes from both NCD-fed *Piezo1^fl/fl^* and *IntL-Piezo1^−/−^* mice treated with EX-4 showed similar results (fig. S3).

**Fig. 6.**
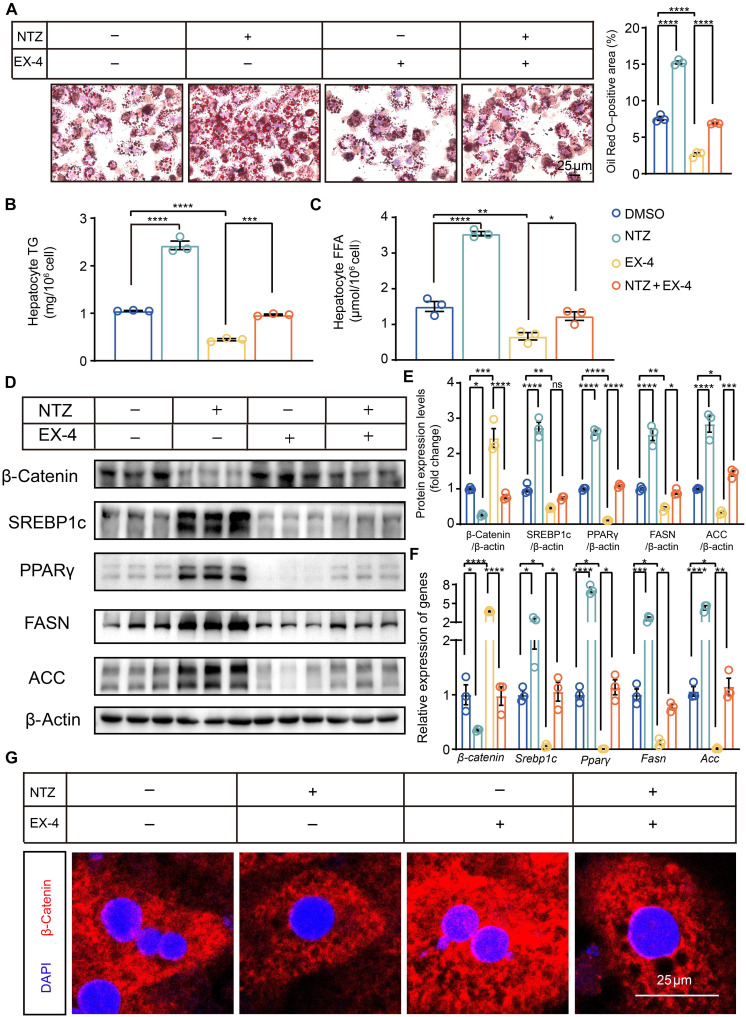
NTZ blocks EX-4’s effect on lipid accumulation in primary hepatocytes. Primary hepatocytes from HFD-fed *IntL-Piezo1^−/−^* mice were treated with dimethyl sulfoxide (DMSO), NTZ (10 μM), EX-4 (100 nM), and NTZ and EX-4. (**A**) Oil Red O–stained pictures of primary hepatocytes. (**B** and **C**) Hepatocyte TG and FFA levels. (**D**) Western blot of whole-cell extracts with the indicated antibodies such as β-catenin, SREBP1c, PPARγ, FASN, ACC. (**E**) Western blot quantification. (**F**) β*-catenin*, *Srebp1c*, *Ppar*γ, *Fasn*, and *Acc* mRNA levels. (**G**) Immunofluorescence staining for β-catenin (red). Data are presented as the means ± SEM and represent three biological replicates. Significance was determined by one-way ANOVA followed by post hoc analysis in (A) to (G). *P < 0.05, **P < 0.01, ***P < 0.001, and ****P < 0.0001.

To investigate whether the action of GLP-1 is mediated by β-catenin, nitazoxanide (NTZ), an inhibitor of β-catenin, was used. NTZ blocked the improvement of fatty acid accumulation induced by EX-4 in primary liver cells of *IntL-Piezo1^−/−^* fed with an HFD. Furthermore, NTZ inhibited the expression and nuclear translocation of β-catenin triggered by EX-4 (Fig. 6, A to G). NTZ plays a similar role in primary liver cells of *IntL-Piezo1^−/−^* fed with a normal diet (fig. S4).

## DISCUSSION

MASLD is a chronic liver disease caused by multiple metabolic disorders (*1*, *25*). Its pathogenesis is a complex and multifactorial process. The key pathological features involve disruptions in lipid metabolism, such as increased fatty acid synthesis and decreased fatty acid oxidation in the liver, ultimately leading to hepatic lipid accumulation (*26*, *27*). This lipid accumulation exacerbates insulin resistance, creating a vicious cycle that further impairs liver function (*28*, *29*). Although the precise cellular mechanisms driving the development of hepatic steatosis have not been fully elucidated, enhanced activity of the lipogenic pathway appears to be a contributing factor. Therefore, targeting the inhibition of de novo lipogenesis has emerged as a potential therapeutic approach for the management of MASLD. In recent years, the gut-hepatic axis has emerged as an important regulator of hepatic lipid metabolism, acting through either neuroreflexes or intestinal hormones (*30*, *31*). Previous studies have shown that the vagus nerve connects the gut and liver, transmitting signals that affect metabolic processes (*32*). Gut hormones such as fibroblast growth factor 15 in mice (or fibroblast growth factor 19 in humans) (*33*), PYY (*31*), ghrelin (*34*), and GLP-1 (*35*) play a role in controlling hepatic glucose and lipid metabolism. GLP-1, secreted by intestinal L cells, is closely linked to lipid metabolism. It can be involved in lipid metabolism by inhibiting fat synthesis (*35*), promoting fat differentiation (*10*), enhancing cholesterol metabolism (*36*), and promoting fat browning (*37*). Piezo1 is a mechanosensitive ion channel that reacts to mechanical forces (*14*, *15*). Our earlier studies demonstrated that Piezo1 can sense mechanical stretching in the gastrointestinal tract, playing a role in the regulation of hormone synthesis and secretion (*23*, *38*). We generated L cell–specific *Piezo1* gene knockout mice, designated as *IntL-Piezo1^−/−^* mice. These mice displayed impaired glucose tolerance and reduced GLP-1 production through the CaMKKII-CaMKIV-mechanistic target of rapamycin (mTOR) pathway (*23*). In addition, as part of another study, we conducted a comprehensive analysis of how mTOR regulates the transcription and translation of the *GCG* gene, which encodes the precursor protein for GLP-1 (*39*). In the current study, we further report that *IntL-Piezo1^−/−^* mice also demonstrated reduced GLP-1 secretion and developed hepatic steatosis. In addition, the levels of hepatic lipogenesis–related transcription factors and enzymes were elevated in *IntL-Piezo1^−/−^* mice compared to control mice, indicating enhanced hepatic lipid synthesis.

In recent years, research has shown that GLP-1R expression is present in human hepatocytes (*40*). However, the presence of GLP-1R in liver tissue continues to be a matter of debate (*41*). Furthermore, it has been demonstrated that GLP-1R agonists act as a regulatory pathway that influences metabolic disorders (*42*). Clinical investigations into the use of GLP-1 agonists for treating liver disease in humans have already progressed to validated phase 2 trials (*43*). In the current study, we observed that EX-4 alleviates hepatic steatosis in *IntL-Piezo1^−/−^* mice, suggesting that the hepatic lipid disorder in these mice may be attributed to a deficiency in GLP-1 production and release. As reported in the literature, GLP-1 is capable of affecting liver lipid metabolism via both central and peripheral pathways. A study has shown that intracerebroventricular infusion of GLP-1 in mice significantly and effectively reduces lipid storage in both white adipose tissue and the liver, independent of nutrient intake. This central nervous system regulation of adipocyte metabolism partially depends on a functioning sympathetic nervous system. In addition, the impact of central GLP-1 on adipocyte and liver metabolism is weakened in mice with diet-induced obesity (*44*). Another research has demonstrated that the GLP-1 receptor agonist liraglutide, when administered subcutaneously, can inhibit hepatic lipogenesis by activating the AMPK/mTOR/SREBP1 signaling pathway (*45*). In addition, the Wnt/β-catenin signaling pathway is critically involved in various metabolic disorders, including hepatic steatosis and insulin resistance (*46*). In HepG2, Huh7, and AML12 cells, the mRNA expression of β-catenin was found to increase in a dose-dependent and time-dependent manner following treatment with EX-4 (*47*). GLP-1 might augment β-catenin levels by stabilizing it, thereby leading to an increased accumulation of β-catenin in the cytoplasm. This accumulation facilitates its translocation to the nucleus, where it activates genes associated with cell growth and metabolism (*48*, *49*). Our study revealed that the expression of β-catenin was significantly reduced in the livers and primary hepatocytes of *IntL-Piezo1^−/−^* mice. This decrease was associated with an increased expression of genes and enzymes related to lipid synthesis when compared to control mice, regardless of whether they were on an HFD or a normal diet.

NTZ is widely recognized as a β-catenin inhibitor in research contexts (*50*). In this study, we found that NTZ treatment significantly reduced the regulatory effects of EX-4 on *IntL-Piezo1^−/−^* hepatocyte lipid metabolism, suggesting that the β-catenin signaling pathway mediates some of the actions of EX-4. To further clarify the essential role of β-catenin in the GLP-1–mediated regulation of hepatocyte lipid metabolism in *IntL-Piezo1^−/−^* mice, we performed nuclear-cytoplasmic fractionation experiments and conducted immunofluorescence analysis. Our results revealed that EX-4, a GLP-1 receptor agonist, notably enhanced the nuclear translocation of β-catenin in primary hepatocytes of both *Piezo1^fl/fl^* and *IntL-Piezo1^−/−^* mice. This finding provides compelling evidence for the molecular mechanism by which GLP-1 regulates hepatic lipid metabolism, potentially through β-catenin. However, a study has indicated that NTZ inhibits the activity of the AKT/mTOR and Wnt/β-catenin signaling pathways in osteosarcoma cells (*51*). As a result, the inhibitory effect of NTZ on β-catenin may not be entirely specific. We cannot fully discount the possibility that it may indirectly modulate other signaling pathways as well.

Notably, the secretion of gut hormones during feeding is regulated not only by the chemical stimulation of nutrients but also by the physical movement of digesta. A previous study has shown that Piezo1 channels in L cells are essential for sensing mechanical stretching, which stimulates the synthesis and secretion of GLP-1 and helps regulate glucose homeostasis (*23*). As food enters the intestine, it causes L cells to stretch, leading to the release of GLP-1, which in turn reduces postprandial hepatic fatty acid synthesis and lipid accumulation. When the mechanical sensing mechanism of L cells is impaired, GLP-1 production is disrupted, resulting in increased hepatic lipid synthesis and the development of fatty liver. Our current research indicates that mechanosensing in L cells affects lipid synthesis in the liver. These findings offer valuable insights into targeting the gut as a strategy for intervening in MASLD.

Gastric balloon implantation is widely regarded as an effective method for treating obesity (*52*). Its mechanism is partly attributed to the balloon occupying space within the stomach, which reduces food intake and enhances feelings of satiety (*52*). Previous studies have indicated that nutrients can strongly inhibit natural or sham feeding in a dose-dependent manner, with inhibitory signals transmitted from the ileum and duodenum. Feedback from the ileum may be more potent than that from more proximal regions of the intestine (*53*–*55*). In this study, we used an HFD-induced C57BL/6J mouse model of fatty liver and found that the implantation of silicone balls in the ileum significantly increased GLP-1 secretion compared to the sham surgery group. This enhancement was closely associated with reduced circulating lipid levels and decreased hepatic lipid accumulation. Moreover, intestinal bead implantation markedly suppressed de novo fatty acid synthesis in the mouse liver. However, in HFD-fed *IntL-Piezo1^−/−^* mice, intestinal bead implantation did not lead to an increase in GLP-1 secretion, nor did it improve hepatic de novo fatty acid synthesis. These findings suggest that intestinal bead implantation may effectively reduce hepatic lipid synthesis by mechanically activating Piezo1 in ileal L cells. Our current study underscores the potential role of mechanical stimulation–induced “intestinal satiety” in the treatment of MASLD.

In summary, our research suggests that manipulating the mechanosensitive ion channel Piezo1 in intestinal L cells can influence the release of GLP-1, with potential implications for the progression of fatty liver disease. These findings provide a theoretical framework for identifying potential targets and treatments for fatty liver disease.

## MATERIALS AND METHODS

### Animals and treatment

The mice were kept in a temperature- and humidity-controlled specific pathogen–free environment with a 12-hour light-dark cycle. Unless stated otherwise, the mice were provided unrestricted access to either an NCD or HFD as well as water. The animal protocols were approved by the Animal Care and Use Committee of Jinan University.

An L cell–specific *Piezo1* knockout mouse model was created using established methods (*23*). Briefly, *IntL-Cre* mice were crossed with *Piezo1^fl/fl^* mice to generate L cell–specific *Piezo1*-deficient (*IntL-Piezo1*^−/−^) mice, which were then genotyped by polymerase chain reaction (PCR).

Male *IntL-Piezo1^−/−^* mice between the ages of 12 to 16 weeks and age-matched control littermates (*Piezo1^fl/fl^* mice) fed with NCD (control diet, D12450H; Research Diets) or HFD (60% fat, D12492; Research Diets) were used in the current study. Male *Piezo1^fl/fl^* and *IntL-Piezo1*^−/−^ mice fed with 10-week HFD were intraperitoneally injected with normal saline (NS) or the GLP-1R agonist EX-4 (100 μg/kg body weight) for 7 consecutive days. Twelve- to 16-week-old male C57BL/6J mice and *IntL-Piezo1^−/−^* mice were fed an HFD and then divided into groups based on whether they underwent sham surgery or intestine bead implantation.

### In situ hybridization

In situ hybridization was conducted as previously described (*23*). Paraffin sections were deparaffinized and rehydrated before undergoing antigen retrieval in citrate buffer (pH 6.0). The sections were then treated with proteinase K (5 μg/ml) at 37°C for 15 min. Subsequently, the sections were subjected to overnight hybridization with probes in a temperature-controlled chamber set at 40°C. The Piezo1 probe sequences used were as follows: 5′-CTGCAGGTGGTTCTGGATATAGCCC-3′, 5′-AAGAAGCAGATCTCCAGCCCGAAT-3′, and 5′-GCCATGGATAGTCAATGCACAGTGC-3′. Following washing with saline-sodium citrate (SSC) buffers, the sections underwent hybridization with prewarmed branch probes at 40°C for 45 min. Another round of washing with SSC buffers was followed by hybridization with the signal probe at 42°C for 3 hours. After further washing with SSC buffers, the sections were blocked with normal serum and then incubated overnight at 4°C with a mouse anti-GLP-1 (1:200) antibody, followed by a secondary antibody. Imaging was conducted using laser scanning confocal microscopy, and fluorescence signals were quantified using ImageJ.

### Intestinal bead implantation

C57BL/6J mice with fatty liver induced by an HFD, as well as *IntL-Piezo1^−/−^* mice on an HFD, were subjected to a fasting period of 6 to 8 hours before the procedure. A 1-cm incision was made on the abdominal wall to expose the intestine. Another 1-cm incision was made about 1 cm above the ileocecal region. A 2.5-mm-diameter bead was then implanted into the ileum through this incision. For the sham operation, all procedures were identical, except that the bead was not implanted.

### Isolation and culture of primary mouse hepatocytes

Primary mouse hepatocytes were isolated using a modified two-step collagenase perfusion technique. The livers were perfused with a Ca^2+^-free Krebs-Henseleit buffer containing collagenase IV (2 mg/ml). Following a 15-min digestion, the hepatocytes were filtered, washed, centrifuged, and resuspended in Dulbecco’s modified Eagle’s medium. After counting the cells, they were plated in collagen-coated six-well plates at a density of 1 × 10^6^ cells/ml and cultured in Dulbecco’s modified Eagle’s medium supplemented with 10% fetal bovine serum.

### Measurement of GLP-1 secretion

Samples were collected in the presence of aprotinin (2 μg/ml), EDTA (1 mg/ml), and diprotinA (0.1 mM) and were stored at −80°C before use. GLP-1 levels were assayed using enzyme immunoassay kits according to the manufacturer’s instructions.

### Hematoxylin and eosin staining

Tissues were collected, fixed with 4% paraformaldehyde, embedded in paraffin, and cut into 4-μm sections. Standard protocols were followed for staining the sections with hematoxylin and eosin (HE). Photomicrographs were captured under an inverted microscope (Leica, Germany).

### Oil Red O staining

The liver was preserved in 4% paraformaldehyde and embedded in optimal cutting temperature compound for histological examination. For Oil Red O staining, 10-μm tissue slices were prepared and stained with Oil Red O. Hematoxylin stained the nuclei, allowing for microscopic observation of red lipid droplets.

### Immunofluorescence

Primary hepatocytes were fixed in 4% paraformaldehyde for 20 min at room temperature and permeabilized with 0.3% Triton X-100 for 5 min. After blocking with 3% bovine serum albumin, the cells were incubated overnight at 4°C with a rabbit anti-β-catenin primary antibody (diluted 1:200). The next day, the cells were washed three times with phosphate-buffered saline. Subsequently, they were incubated with a DyLight 594 AffiniPure donkey anti-rabbit immunoglobulin G (IgG) antibody (diluted at a ratio of 1:100) for 1 hour at room temperature. Nuclei were counterstained with DAPI (4′,6-diamidino-2-phenylindole). Images were captured using a laser scanning confocal microscope (Leica SP8).

### Biochemical analysis

Free fatty acid (FFA) and triglyceride (TG) levels in blood and liver were measured using commercial kits (boxbio, Shanghai, China) according to the instructions.

### Western blot analysis

For the Western blot experiments, tissues and cells were harvested, and total proteins were extracted using radioimmunoprecipitation assay buffer supplemented with a cocktail of protease and phosphatase inhibitors. Nuclear and cytoplasmic fractions of primary hepatocyte proteins were isolated using a nuclear protein extraction kit (Beyotime, cat. no. P0028) with the addition of phenylmethylsulfonyl fluoride. Subsequently, protein concentrations were determined. Proteins were separated using SDS–polyacrylamide gel electrophoresis and subsequently transferred to a nitrocellulose membrane. The membrane was blocked at room temperature for 1 hour with 5% skimmed milk prepared in 1× tris-buffered saline with Tween 20 [TBST; consisting of 25 mM tris, 150 mM NaCl, 2 mM KCl (pH 7.4), and 0.2% Tween 20]. Following this, the membrane was incubated overnight at 4°C with the following primary antibodies: anti-β-Catenin (1:1000), anti-SREBP1c (1:1000), anti-PPARγ (1:1000), anti-fatty acid synthase carboxylase (1:1000), anti-acetyl-CoA carboxylase (1:1000), anti-β-actin (1:5000), and anti-Lamin A/C (1:1000). The following day, the membrane was washed three times with TBST, each wash lasting 10 min. It was then incubated at room temperature for 1 hour with horseradish peroxidase–conjugated goat anti-mouse IgG (H + L) (1:10000) or horseradish peroxidase–conjugated goat anti-rabbit IgG (H + L) (1:5000) secondary antibodies. Afterward, the membrane was washed three additional times with TBST. Protein bands were visualized using luminol reagent, and their intensities were quantified with background subtraction using ImageJ software.

### Real-time PCR

Total RNA was extracted with TRIzol reagent (Takara, Japan) and converted to cDNA through reverse transcription. Quantitative PCR was carried out using SYBR Green Master Mix (Vazyme, Nanjing, China) following the manufacturer’s guidelines. The primers used are detailed in table S1.

### Key resources

Key reagents or resources are listed in table S2.

### Statistical analysis

All data were expressed as the means ± SEM. The significance of differences was determined by Student’s *t* test or one-way analysis of variance (ANOVA) followed by post hoc analysis. *P* values less than 0.05 were considered statistically significant (*P < 0.05, **P < 0.01, ***P < 0.001, and ****P < 0.0001; ns, not significant). The research team was not informed of the experimental conditions throughout the biochemical and imaging studies. A randomization scheme was implemented to assign mice with identical genetic backgrounds to various experimental groups during drug administration and surgical procedures. All data were included in the analysis.
