# Supplementary material for: Intestinal L-cell mechanoreception regulates hepatic lipid metabolism through GLP-1
Source: Sci Adv. 2025 May 30;11(22):eadv3201. doi: 10.1126/sciadv.adv3201 (PMC12124353; doi:10.1126/sciadv.adv3201)
Supplement: Supplementary file 1 — Figs. S1 to S4 Tables S1 and S2 [file sciadv.adv3201_sm.pdf]

Science Advances
AAAS

Supplementary Materials for

Intestinal L-cell mechanoreception regulates hepatic lipid metabolism through GLP-1

Luyang Gao *et al.*

Corresponding author: Geyang Xu, xugeyangliang@163.com

Sci. Adv. **11**, eadv3201 (2025)
DOI: 10.1126/sciadv.adv3201

This PDF file includes:

Figs. S1 to S4
Tables S1 and S2

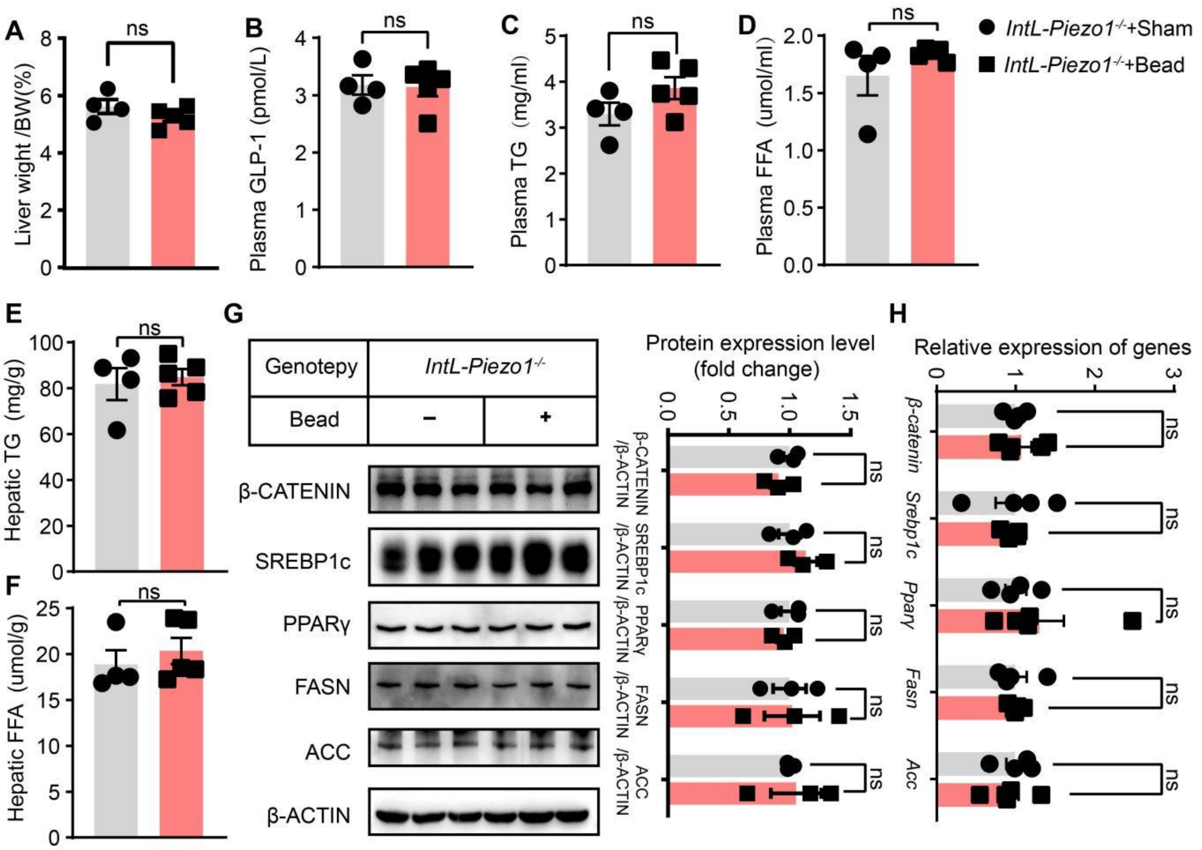

Fig. S1. Silicone beads in intestines of *IntL-Piezo1*$^{-/-}$ mice do not affect liver lipids
(**A**) The liver-to-body weight ratio of *IntL-Piezo1*$^{-/-}$ mice that underwent sham surgery or intestinal bead implantation (n = 4 or 5 per group). (**B**) Plasma GLP-1 levels, (**C**) plasma triglycerides (TG), (**D**) plasma free fatty acids (FFA), (**E**) hepatic TG, and (**F**) hepatic FFA. (**G**) Western blot analysis was performed to measure the expression of β-CATENIN, sterol regulatory element-binding protein 1c (SREBP1c), peroxisome proliferator-activated receptor γ (PPARγ), fatty acid synthase (FASN), and acetyl-CoA carboxylase (ACC) in the liver of *IntL-Piezo1*$^{-/-}$ mice that underwent sham surgery or intestinal bead implantation. (**H**) Hepatic mRNA levels of *β-catenin*, *Srebp1c*, *Pparγ*, *Fasn*, *Acc*. Data in [(**A**) to (**H**)] are presented as mean ± SEM from four or five biological replicates. Student's t-test was used to determine statistical significance in [(**A**) to (**H**)], n=4 or 5, ns = no significance, *$p < 0.05$, **$p < 0.01$, ***$p < 0.001$, ****$p < 0.0001$.

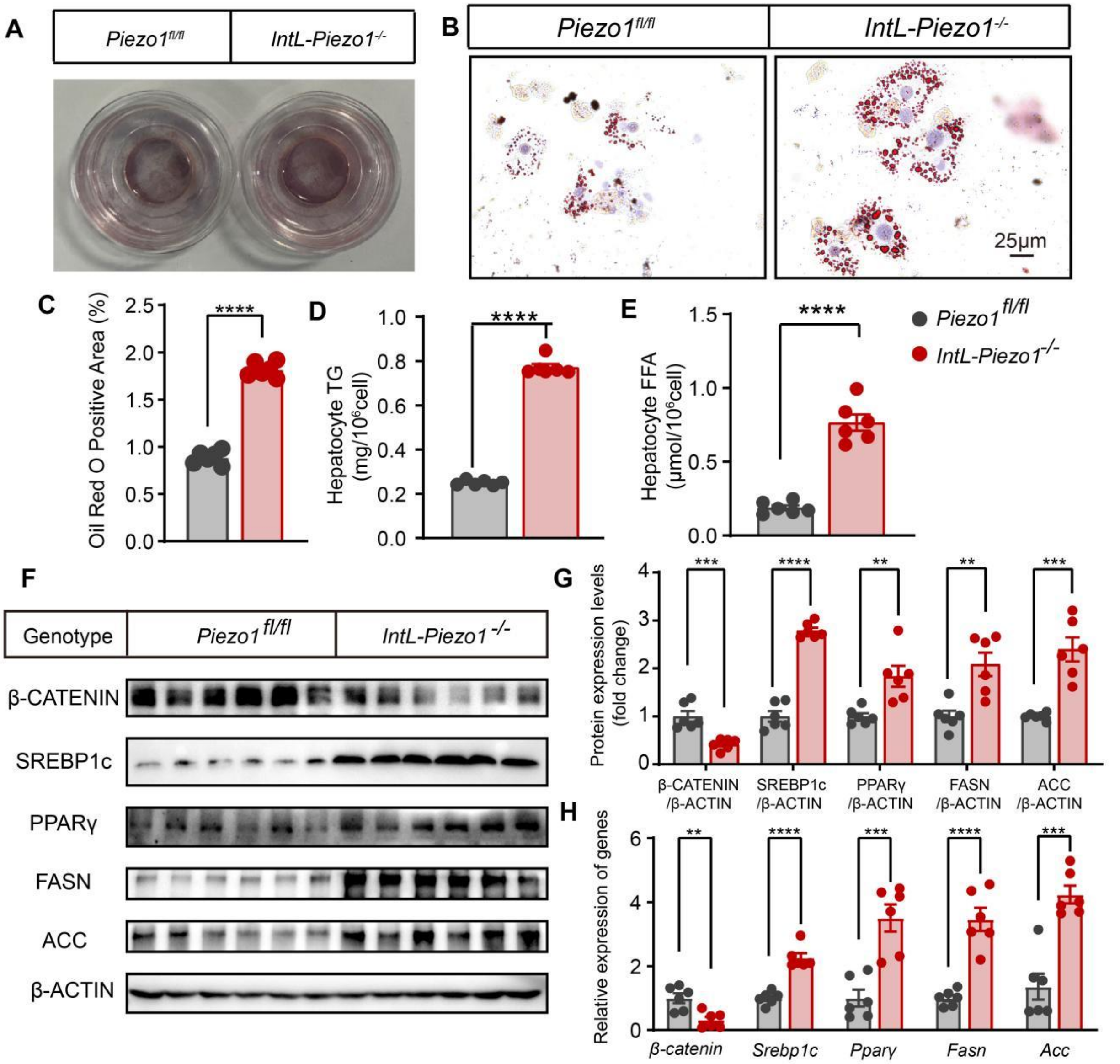

Fig. S2. Primary hepatocytes from NCD-fed *IntL-Piezo1*$^{-/-}$ mice exhibit lipid accumulation
(**A** and **B**) Oil Red O staining images of primary hepatocytes from *Piezo1*$^{fl/fl}$ and *IntL-Piezo1*$^{-/-}$ mice under NCD. (**C**) Quantification of Oil Red O staining. (**D** and **E**) Hepatocyte TG and FFA levels. (**F**) Western blot analysis of whole cell extracts using antibodies against β-CATENIN, SREBP1c, PPARγ, FASN, and ACC. (**G**) Quantification of western blot. (**H**) mRNA levels of *β-catenin*, *Srebp1c*, *Pparγ*, *Fasn*, *Acc* in primary hepatocytes from *Piezo1*$^{fl/fl}$ and *IntL-Piezo1*$^{-/-}$ under NCD. Data are represented as mean ± SEM. Significance was determined by Student's t test. n=6, *p < 0.05, **p < 0.01, ***p < 0.001, ****p < 0.0001.

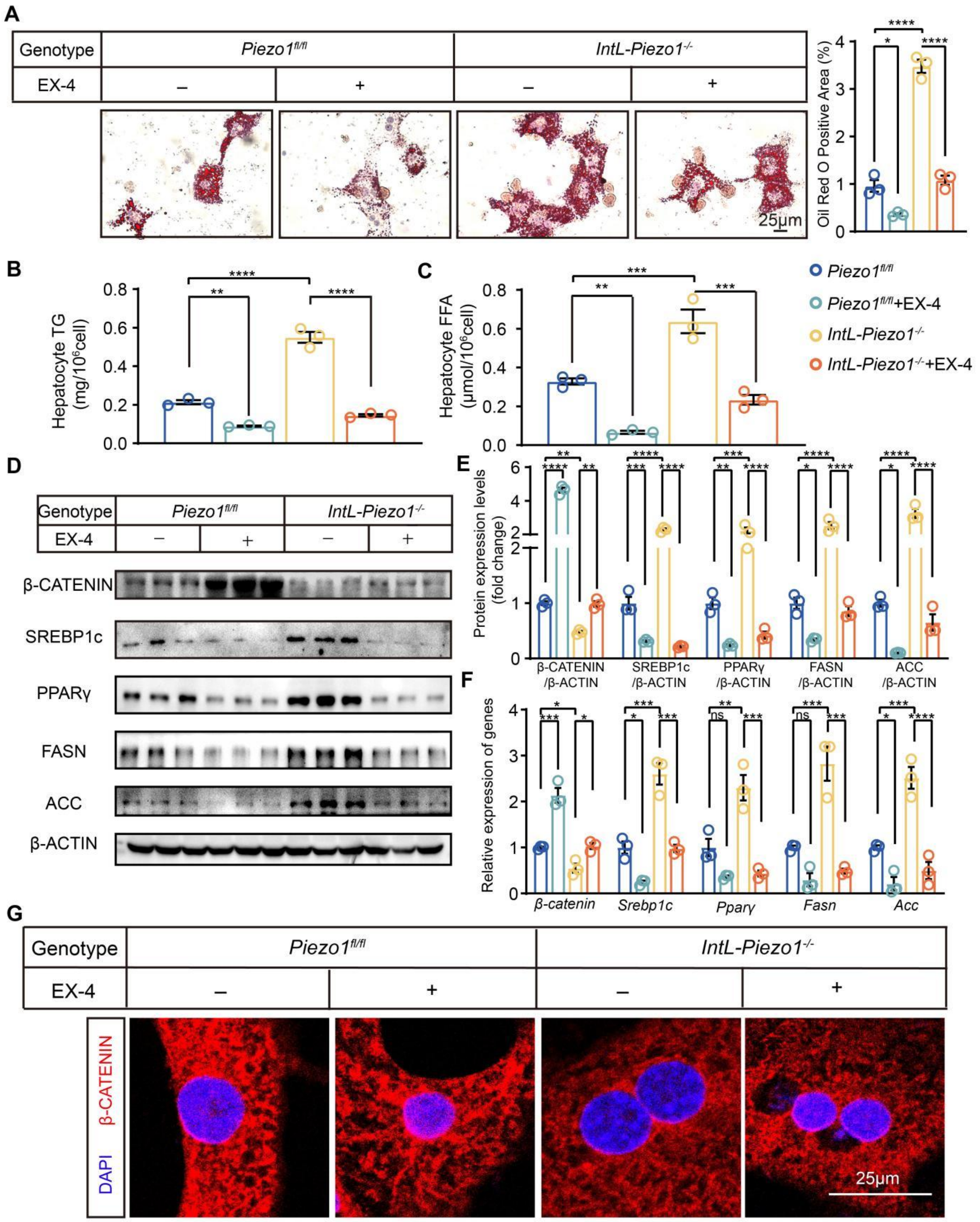

Fig. S3. EX-4 reduces lipid accumulation in primary hepatocytes of NCD-fed *IntL-Piezo1$^{-/-}$* (**A**) Oil red O-stained pictures of primary hepatocytes from normal chow diet fed *Piezo1$^{fl/fl}$* and *IntL-Piezo1$^{-/-}$* mice after EX-4 (100nM) treatment for 24 hours. (**B** and **C**) Hepatocyte TG and FFA levels. (**D**) Western blot of whole cell extracts with the indicated antibodies such as β-

CATENIN, SREBP1c, PPARγ, FASN, ACC. (**E**) Western blot quantification. (**F**) *β-catenin, Srebp1c, Pparγ, Fasn and Acc* mRNA levels in primary hepatocytes from normal chow diet fed *Piezo1*$^{fl/fl}$ and *IntL-Piezo1*$^{-/-}$ mice after EX-4 (100nM) treatment for 24 hours.
(**G**) Immunofluorescence staining for β-catenin (red) in primary hepatocytes from normal chow diet fed *Piezo1*$^{fl/fl}$ and *IntL-Piezo1*$^{-/-}$ mice after EX-4 treatment for 24 hours. Data are presented as mean ± SEM and represent three biological replicates. One-way ANOVA was used for comparison among the four groups followed by post-hoc analysis, $*p < 0.05$, $**p < 0.01$, $***p < 0.001$, $****p < 0.0001$.

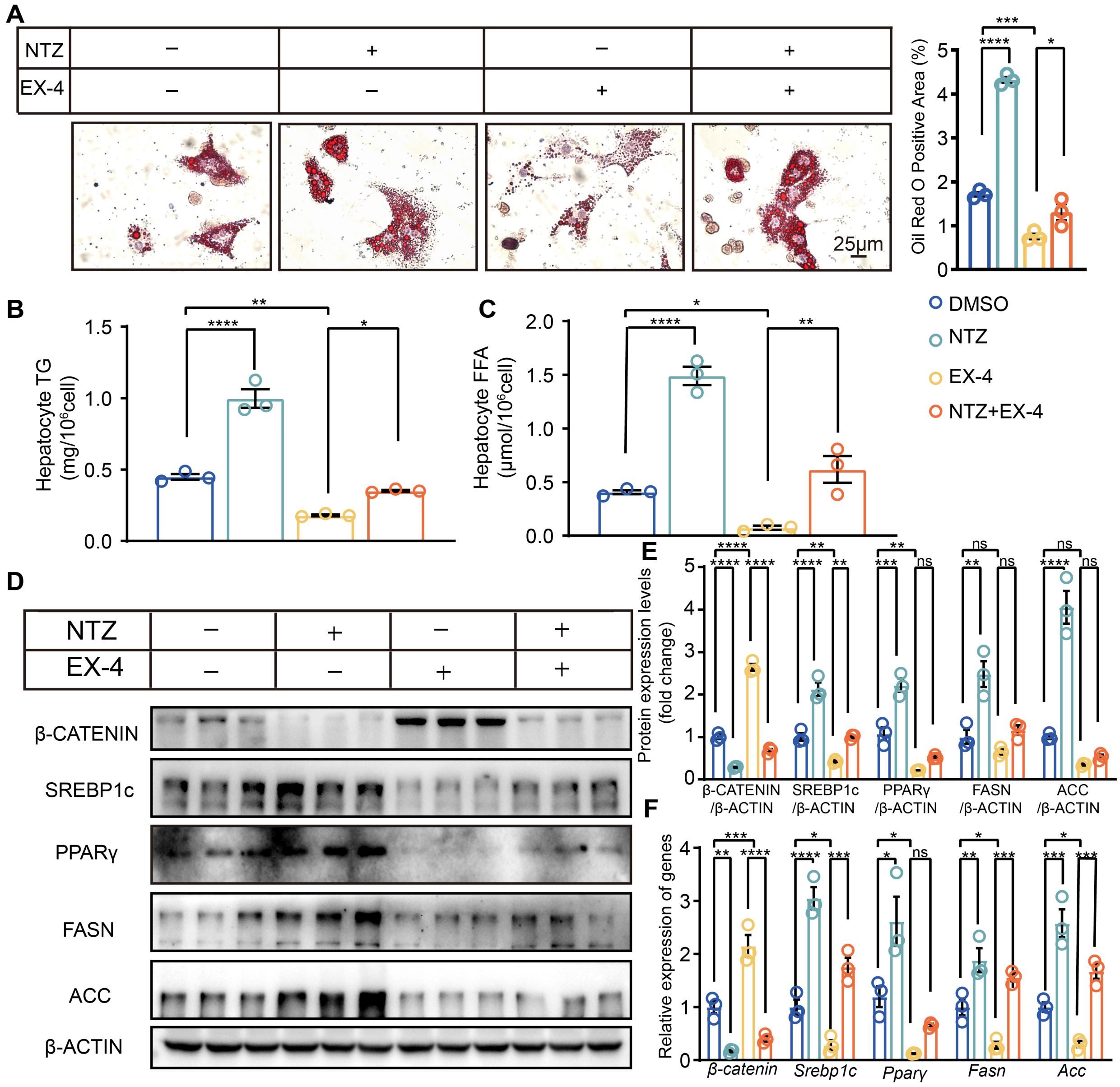

Fig. S4. Nitazoxanide inhibits EX-4's effect on lipid metabolism in *IntL-Piezo1*$^{-/-}$ hepatocytes
Primary hepatocytes from NCD-fed *IntL-Piezo1*$^{-/-}$ mice were treated with DMSO, Nitazoxanide (NTZ, 10μM), EX-4 (100 nM), NTZ plus EX-4. (**A**) Oil red O-stained pictures of primary hepatocytes. (**B** and **C**) Hepatocyte TG and FFA levels. (**D**) Western blot of whole cell extracts with the indicated antibodies such as β-CATENIN, SREBP1c, PPARγ, FASN, ACC. (**E**) Western blot quantification. (**F**) *β-catenin, Srebp1c, Pparγ, Fasn and Acc* mRNA levels. Data are presented as mean ± SEM and represent three biological replicates. One-way ANOVA was used for comparison among the four groups followed by post hoc analysis, ns = no significance, **p* < 0.05, ***p* < 0.01, ****p* < 0.001, *****p* < 0.0001.

Table S1. Sequences of primers used in RT-PCR experiments, Related to method details

	Upstream primer (5'-3')	Downstream primer (5'-3')	Accession
β-catenin	TGCTCATCCCACTAATGTCC	AGCTTCCTTTTTGGAAAGCT	NM_001165902
Srebp1c	GGAGCCATGGATTGCACATT	GGAAGTCACTGTCTTGGTTG	XM_006532716
Pparγ	TCAGCTCTGTGGACCTCTCC	ACCCTTGCATCCTTCACAAG	XM_017321456
Fasn	TGGGTTCTAGCCAGCAGAGT	ACCACCAGAGACCGTTATGC	NM_007988.3
Acc	TGGTCGTGACTGCTCTGTGC	GTAGCCGAGGGTTCAGTTCC	XM_006531957
β-actin	CCACAGCTGAGAGGGAAAT	AAGGAAGGCTGGAAAAGAG	NM_007393.5

Table S2. Key resources table

REAGENT or RESOURCE	SOURCE	IDENTIFIER
Antibodies		
Rabbit anti-β-Catenin (WB:1:1000,IF:1:200)	Affinity	Cat#AF6266,RRID:AB_2835124
Mouse anti-SREBP1 [2A4] (WB: 1:1000)	Abcam	Cat# ab3259, RRID:AB_303650
Rabbit anti-PPAR (WB: 1:1000)	Proteintech	Cat# 16643-1-AP, RRID:AB_10596794
Rabbit anti-fatty acid synthase carboxylase (WB: 1:1000)	Abcam	Cat# ab99359, RRID:AB_10697253
Rabbit anti-acetyl-CoA carboxylase (WB: 1:1000)	Cell Signaling Technology	Cat# 3676, RRID:AB_2219397
Mouse anti-β-actin (WB: 1:1000)	Cell Signaling Technology	Cat# 3700, RRID:AB_2242334
Rabbit anti-LaminA/C (WB: 1:1000)	Cell Signaling Technology	Cat# 2032, RRID:AB_2136278
Dylight 594 affinipure donkey anti-rabbit IgG	EarthOx LLC	Cat# E032421-01
Horseradish peroxidase-conjugated, Goat Anti-Rabbit IgG	Jackson ImmunoResearch Labs	Cat# 111-035-003, RRID:AB_2313567
Horseradish peroxidase-conjugated, Goat Anti-Mouse IgG	Jackson ImmunoResearch Labs	Cat# 115-035-003, RRID:AB_10015289
Reagents and kits		
0.1% gelatine	Biological Industries	Cat# 01-944-1B
DMEM high sugar medium	Gibco	Cat# 11965092
Fetal bovine serum	Gibco	Cat# 12484028
Immobilon western chemiluminescent HRP substrate	Millipore	Cat# WBKLS0500
Diprotin A	Sigma-Aldrich	Cat# 90614-48-5
RT-PCR kit	Takara	Cat# RR014A
Nuclear and Cytoplasmic Protein Extraction Kit	Beyotime	Cat# P0028
Thermo Scientific TurboFect Transfection Reagent	Thermo Fisher Scientific	Cat# R0531
TRIzol	Thermo Fisher Scientific	Cat# 15596026
RIPA Lysis Buffer	Beyotime Biotechnology	Cat# P0013B
Chemicals, peptides, and recombinant proteins		
Exendin-4	Sigma-Aldrich	Cat# E7144
Nitazoxanide	MedChemExpress	Cat# 55981-09-4
Oligonucleotides		
Primers for cloning and RT-PCR, see Tables S1 and S2	This paper	N/A
Other		
Normal chow diet	Research Diets	Cat# D12450B
High fat diet	Research Diets	Cat# D12492
